# Repurposing Asparaginase Therapy to Target Cisplatin‐Resistant Cancer Cells

**DOI:** 10.1111/fcp.70044

**Published:** 2025-08-10

**Authors:** Jiantao Wang, Nasim Pouryaghoub, Robert Strauss, Jiri Bartek, Si Min Zhang, Sean G. Rudd

**Affiliations:** ^1^ Science for Life Laboratory (SciLifeLab), Department of Oncology‐Pathology, Karolinska Institutet Stockholm Sweden; ^2^ Department of Radiation Oncology, West China Hospital Sichuan University Chengdu China; ^3^ Lung Cancer Center, West China Hospital Sichuan University Chengdu China; ^4^ Danish Cancer Institute Genome Integrity Group Copenhagen Denmark; ^5^ Science for Life Laboratory (SciLifeLab), Division of Genome Biology, Department of Medical Biochemistry and Biophysics Karolinska Institute Stockholm Sweden

**Keywords:** asparaginase (ASNase), chemoresistance, cisplatin, glutamine, metabolic rewiring, SLC7A11

## Abstract

**Background:**

Cisplatin and its derivatives remain a cornerstone in the treatment of solid malignancies. Resistance is a major factor limiting their clinical utility.

**Objectives:**

In the present study, we set out to interrogate therapeutic approaches to target cisplatin‐resistant cancer cells. We focused on therapies exploiting metabolic pathways that are altered in drug‐resistant cells. We sought to find an existing therapy that has monotherapy efficacy against cisplatin‐resistant cancer cells that can also re‐sensitize to cisplatin.

**Methods:**

We used lung and ovarian cancer cell lines with acquired resistance to cisplatin together with drug sensitivity assays, conducted both with monotherapies and cisplatin combinations.

**Results:**

We show that cancer cell lines with acquired resistance to cisplatin have altered levels of enzymes involved in glutamine metabolism, which can result in differential sensitivity to targeted agents. We show that expression of one of these enzymes—the glutamate‐cystine antiporter SLC7A11, up‐regulated 6‐fold in a cisplatin‐resistant lung cancer cell line—has potential prognostic significance in lung cancer but not ovarian cancer. After identifying a common dependency of cisplatin‐resistant cancer cells upon extracellular glutamine, we then evaluate the utility of the long‐standing anti‐leukemic therapy asparaginase (ASNase)—which possesses both asparaginase and glutaminase activity—as a potential approach. We show ASNase preferentially inhibits the proliferation of cisplatin‐resistant cancer cells and can potentially re‐sensitize these cells to cisplatin.

**Conclusions:**

Our results underpin the prevalence of altered metabolism in cisplatin‐resistant cells and highlight the potential utility of re‐purposing ASNase to target these cells, warranting further investigation.

**Abbreviations**
ASNase: asparaginase
CDDP: cisplatin
GLS: glutaminase
GS: glutamine synthetase
GSH: glutathione
HSA: highest single agent
SLC7A11: solute carrier family 7 member 11
ZIP: zero interaction potency

## 1 Introduction

Conventional chemotherapeutic agents remain a fundamental treatment modality for patients with cancer [1]. The platinum derivative cisplatin (cis‐diamminedichloroplatinum; CDDP) is a cornerstone compound effective in the treatment of a variety of solid malignancies, including cancers of the lung, bladder, and ovaries, amongst others. While a good initial response is common, the duration of this response varies greatly and eventual disease relapse can occur, which is a major clinical problem [2]. Thus, identification of actionable therapeutic strategies to target cisplatin‐resistant cancer cells is vital.

The molecular mode‐of‐action of cisplatin and platinum‐derived compounds is principally understood to center around crosslinking of DNA strands, which are cytotoxic DNA lesions that prevent essential metabolic processes such as DNA replication and transcription [3, 4]. To do this, cisplatin, a prodrug, is first activated inside cancer cells by aquation, which occurs spontaneously in the cytoplasm owing to lower intracellular chloride concentrations compared to the extracellular milieu. This produces a potent electrophile that can react with biological molecules in both the cytosol and nucleus, including the bases in the DNA duplex [3, 4]. Following cytotoxic DNA damage, DNA damage signaling will promote induction of the mitochondrial apoptotic pathway resulting in cell death, which can also be supported by cytosolic targets of aquated‐cisplatin [5]. Cisplatin can be limited by its severe side effects, primarily due to its lack of specificity for tumor cells. In its derivative, carboplatin, the chloride ligands are replaced with a more stable dicarboxylate group, resulting in a slower activation rate and reduced toxicity. However, the DNA‐binding component remains unchanged [3]. Oxaliplatin, another derivative, introduces further structural modifications that lead to the formation of bulkier DNA adducts, which can evade some resistance mechanisms and enhance anti‐cancer activity [3].

Given this multi‐step mode of action of cisplatin and platinum‐derived agents, multiple resistance mechanisms exist, which have been widely documented in both the clinical and preclinical settings [2, 3, 5, 6, 7]. These include reduced drug uptake or increased drug efflux, elevated DNA repair proficiency to remove cytotoxic cisplatin adducts, or, alternatively, increased tolerance of cisplatin adducts and failure to induce apoptotic cell death. In addition, aquated cisplatin can be detoxified from cells by reacting with thiol‐containing molecules, such as glutathione (GSH).

GSH is a tripeptide consisting of 𝛾‐glutamate, cysteine, and glycine, with a major role in maintaining redox homeostasis in cells by reacting with harmful by‐products of aerobic metabolism [8]. Owing to the nucleophile thiol residue which lacks steric hindrance and is highly abundant inside cells, the thiol in GSH can react with electrophiles such as aquated‐cisplatin, thereby protecting cells against this cytotoxic compound. The cysteine component of GSH is generated from cystine, which is imported by antiporter SLC7A11 at the expense of exporting glutamate. The glutamate component of GSH is derived from glutamine, a non‐essential amino acid that is highly abundant in the human body [9]. Glutamine can be obtained through diet and can also be synthesized inside cells by glutamine synthetase (GS). Extracellular glutamine is imported into cells by soluble carrier transporters such as ASCT2 (SLC1A5). Within cells, glutamine is involved in many metabolic processes, including nucleotide biosynthesis, energy production via the TCA cycle, and generation of the antioxidant GSH to maintain redox homeostasis [9].

In the present study, we sought to evaluate therapeutic approaches to target cisplatin‐resistant cancer cells. Using cell models with acquired resistance to this agent, we observed that cisplatin resistance was accompanied by changes in glutamine metabolic enzymes that can lead to differential sensitivity to targeted agents. We identify a dependency of cisplatin‐resistant cells upon extracellular glutamine and subsequently evaluate the ability of the approved anti‐leukemic therapy asparaginase (ASNase) to target these cells.

## 2 Materials and Methods

### 2.1 Cell Lines

Human lung adenocarcinoma cell line HCC4006 was authenticated (Microsynth) and thereafter cultured in escalating doses of clinical‐grade cisplatin (#146262, Hospira Nordic AB) to generate a cell model with acquired resistance. The starting dose of 1 μM cisplatin was raised to 2.5 μM and thereafter 5 μM over a time course of 3 months. HCC4006 cisplatin‐resistant cells were subsequently cultured in 5 μM cisplatin for 3 further months. Human ovarian cancer A2780 and cisplatin‐resistant derivative A2780cis were from the European Collection of Authenticated Cell Cultures (ECACC, Sigma‐Aldrich). All cell lines were cultured in RPMI 1640 GlutaMAX supplemented with 10% heat‐inactivated fetal bovine serum (FBS) and penicillin–streptomycin (100 U/mL and 100 μg/mL, respectively). Culture medium was purchased from ThermoFisher Scientific. Glutamine withdrawal experiments were conducted with glutamine‐free RPMI 1640 medium (#21870076, ThermoFisher Scientific). Cells were grown at 37 °C in 5% CO_2_ humidified incubators, and cultures were regularly monitored and tested negative for the presence of mycoplasma using a commercial biochemical test (MycoAlert, Lonza).

### 2.2 Drugs

Cisplatin (CDDP; Sigma‐Aldrich, #P4394) was prepared in 2 mM stock in water or PBS and stored at 4 °C. Asparaginase (ASNase, Sigma‐Aldrich, #A3809) was prepared in water to a stock concentration of 500 U/mL and stored at −20 °C. Erastin (Sigma‐Aldrich, #E7781), RSL‐3 (Sigma‐Aldrich, #SML2234), and Telaglenastat (CB‐839, MedChemExpress, #HY‐12248) were prepared in DMSO at a concentration of 10 mM and stored at −20 °C.

### 2.3 Transfections

Transfections were performed using INTERFERin (Polyplus Transfection). SLC7A11‐targeting siRNA (Hs_SLC7A11_2 FlexiTube siRNA, #SI00104902, Qiagen) and control siRNA (All Stars Negative Control, Qiagen) were transfected at a 10 nM final concentration.

### 2.4 Immunoblots

Cells were scraped in lysis buffer (50 mM Tris–HCl pH 8, 150 mM NaCl, 1 mM EDTA, 1% Triton X‐100, 0.1% SDS) supplemented with cOmplete EDTA‐free protease inhibitor cocktail (Roche) and Halt phosphatase inhibitor cocktail (Thermo Fisher). Samples were incubated on ice for 30 min to 1 h with occasional vortexing before centrifugation to pellet insoluble material. The Pierce BCA Protein Assay Kit (Thermo Fisher) was used to determine the concentration of the remaining soluble fraction, and samples with equal total protein quantity were prepared with Laemmli Sample Buffer (Bio‐Rad) before denaturation at 95 °C for 5 min. SDS–PAGE was performed using a Bio‐Rad setup with Criterion TGX 4–20% gels (Bio‐Rad) and proteins were transferred to a nitrocellulose membrane using Trans‐Blot Turbo Transfer System (Bio‐Rad), all according to the manufacturer's instructions. Membranes were blocked in Odyssey Blocking Buffer (Li‐Cor) and probed with primary and then species‐appropriate IRDye‐conjugated secondary antibodies (Li‐Cor) before visualization on an Odyssey Fc Imaging System (Li‐Cor). Densitometry analyses were performed using ImageStudio Software (version 6.0, Li‐Cor), and values were normalized to a loading control. Primary antibodies used in this study: xCT/SLC7A11 (Cell Signaling, #12691S, 1:1000), GLS1 (abcam, #ab156876, 1:2000), GLS2 (abcam, #ab113509, 1:1000), GS (abcam, #ab176562, 1:1000), ASCT2 (Sigma‐Aldrich, #ABN73, 1:1000), and α‐tubulin (abcam, #ab7291: 1:5000).

### 2.5 Proliferation Inhibition Assays

Inhibition of cell proliferation by monotherapy or combination treatment was determined by resazurin reduction assay as previously described [10]. Compounds prepared either in DMSO (erastin, RSL‐3, CB‐839; 10 mM) or 0.3% Tween‐20 (CDDP, 2 mM; ASNase, 250 U/mL) were dispensed in dilution series into clear bottomed 384‐well plates (#3764, Corning) using the D300e digital dispenser (Tecan). Solvents were normalized across each plate. Cell suspensions were prepared and dispensed using a MultiDrop (Thermo Fisher). Plates were incubated at 37 °C and 5% CO_2_ for 72 h in a humidity chamber until resazurin (#R17017, Sigma‐Aldrich) dissolved in PBS was added to a final concentration of 0.01 mg/mL. Fluorescence at 530/590 nm (ex/em) was measured after 4–6 h resazurin incubation with Hidex Sense Microplate Reader. Fluorescence intensity for each well was normalized to the average of control wells on the same plate containing cells with solvent (100% viability control) and medium with solvent (0% viability control). The data were analyzed using a four‐parameter logistic model in Prism 10 (GraphPad Software). For calculation of area under the dose–response data, the drug concentration values were log_10_ transformed, and the AUC was calculated in Prism 10 (GraphPad Software). For determination of drug combination effects, relative cell viability measurements across a drug matrix were analyzed using SynergyFinder+ [11, 12] with the zero interaction potency (ZIP), highest single agent (HSA) and Bliss metrics. Synergy summary scores were derived from the average of the synergy scores across the entire dose–response landscape.

### 2.6 Colony Outgrowth Assay

Cells (100–200 per well) were seeded into six‐well plates. To assess glutamine dependence, cells were seeded in glutamine‐free media (#21870076, ThermoFisher Scientific). To assess ASNase sensitivity, cells were seeded in media supplemented with ASNase. Cells were then incubated for 12–14 days, during which media with or without ASNase supplementation was replaced every 3–4 days. Following incubation, media were removed, and the wells were washed carefully in PBS before the colonies were stained with 4% methylene blue (#M9140, Sigma‐Aldrich, Sweden) in methanol for 20 min.

### 2.7 Survival Analyses

Overall survival (OS) analyses for *SLC7A11* expression in lung adenocarcinoma (LUAD) and ovarian serous cystadenocarcinoma (OV) were performed using the Gene Expression Profiling Interactive Analysis tool (GEPIA; http://gepia.cancer‐pku.cn), a web‐based platform integrating RNA‐seq expression data from TCGA and GTEx [13]. The “Survival Analysis” module was used with default settings, applying median expression as the cutoff to stratify high vs. low expression cohorts. Kaplan–Meier (KM) plots and hazard ratios (HR) with 95% confidence intervals (CI) were generated automatically by GEPIA. Log‐rank *p* values were used to assess statistical significance.

In addition, the KM‐Plotter tool (https://kmplot.com/) for lung [14] and ovarian [15] cancer was used. The default settings were selected, analyzing all cohorts and applying a median expression cutoff to stratify high vs. low expression. KM plots and HR values were generated automatically, and log‐rank *p* values were used to assess statistical significance.

### 2.8 Statistical Analyses

Statistical analyses were conducted using GraphPad Prism 10 software. Specific statistical test details are indicated in the corresponding figure legends. Statistical significance is defined as *p* < 0.05, unless otherwise stated. Asterisks in figures signify statistical significance (*p ≤ 0.05, **p ≤ 0.01, ***p ≤ 0.001, ****p ≤ 0.0001).

## 3 Results

### 3.1 Acquired Resistance to Cisplatin Can Lead to Changes in Glutamine Metabolic Enzymes

**FIGURE 1:**
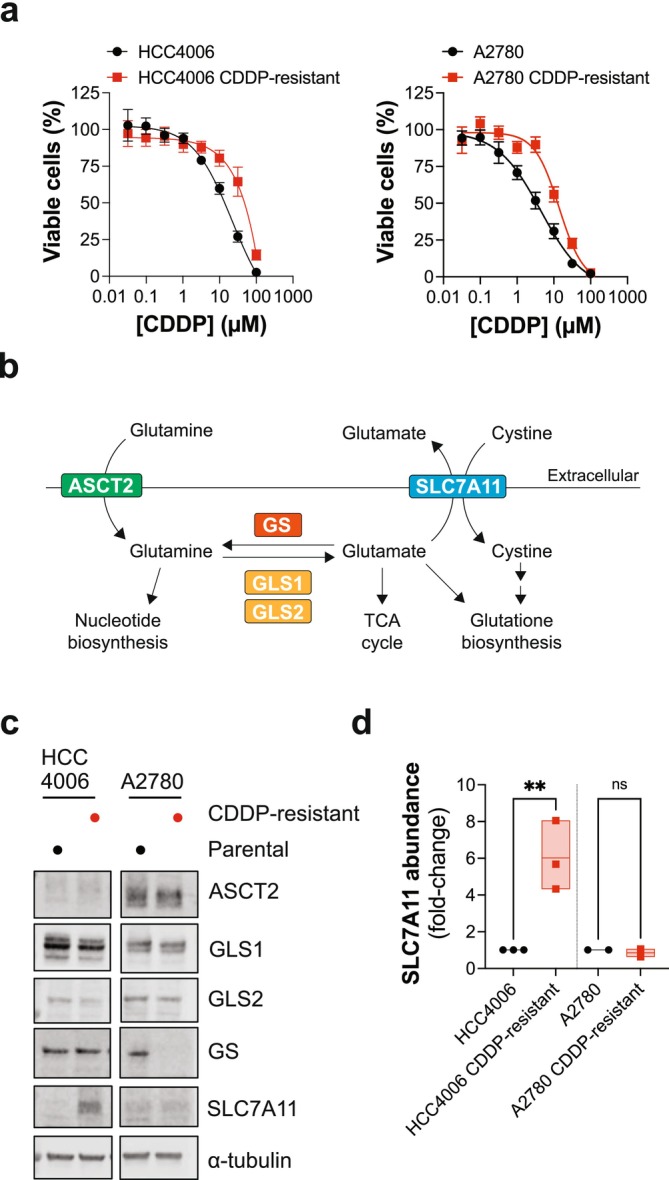
Acquired resistance to cisplatin can lead to altered expression of enzymes involved in glutamine metabolism. (a) Parental and cisplatin (CDDP)‐resistant cell line pairs were exposed to a CDDP titration (100, 31.6, 9.98, 3.17, 1.03, 0.32, 0.1, 0.03 μM) and cell viability measured after 3 days via resazurin reduction. Mean values of three independent experiments shown, each performed with technical duplicates. Error bars indicate SEM. (b) Schematic of glutamine metabolism. ASCT2, glutamine importer; GLS1 and 2, glutaminase 1 and 2; GS, glutamine synthetase; SLC7A11, cystine glutamate antiporter. (c) Immunoblot analysis of lysates prepared from parental and CDDP‐resistant pairs with the indicated antibodies. A representative cropped blot from two independent experiments shown. (d) Densitometry analysis of SLC7A11 protein abundance in the CDDP‐resistant cell lines plotted relative to their parental counterpart. Values from three (HCC4006 cell line pair) or two (A2780 cell line pair) independent experiments plotted, with mean and minimum/maximum values indicated. Ordinary one‐way ANOVA: **p < 0.01; ns, not significant.

To conduct our study, we used cancer cell lines with acquired resistance to cisplatin derived from the lung adenocarcinoma cell line HCC4006 and the ovarian cancer cell line A2780. The parental cell lines and their cisplatin‐resistant derivatives were subjected to a 3‐day incubation of a cisplatin dose–response in a proliferation inhibition assay, which confirmed acquired resistance to this therapy (Figure 1a). Next, we performed immunoblot analysis of lysates from the cell line pairs to determine the protein levels of enzymes involved in glutamine metabolism (Figure 1b,c). Glutamine importer ASCT2 and glutaminases GLS1/2 were expressed in the parental and cisplatin‐resistant cell line pairs; however, glutamine synthetase (GS) and the glutamate‐cystine antiporter SLC7A11 showed strong differential expression (Figure 1c). Specifically, cisplatin‐resistant A2780 cell lines had no detectable GS protein, while cisplatin‐resistant HCC4006 had elevated protein levels of SLC7A11 (Figure 1c), approximately 6‐fold (Figure 1d).

### 3.2 Acquired Resistance to Cisplatin Can Lead to Differential Sensitivity to Targeted Agents

We next investigated the utility of treatment interventions to target the cisplatin‐resistant cancer cells, and focused on treatments targeting glutamine metabolism or downstream processes.

**FIGURE 2:**
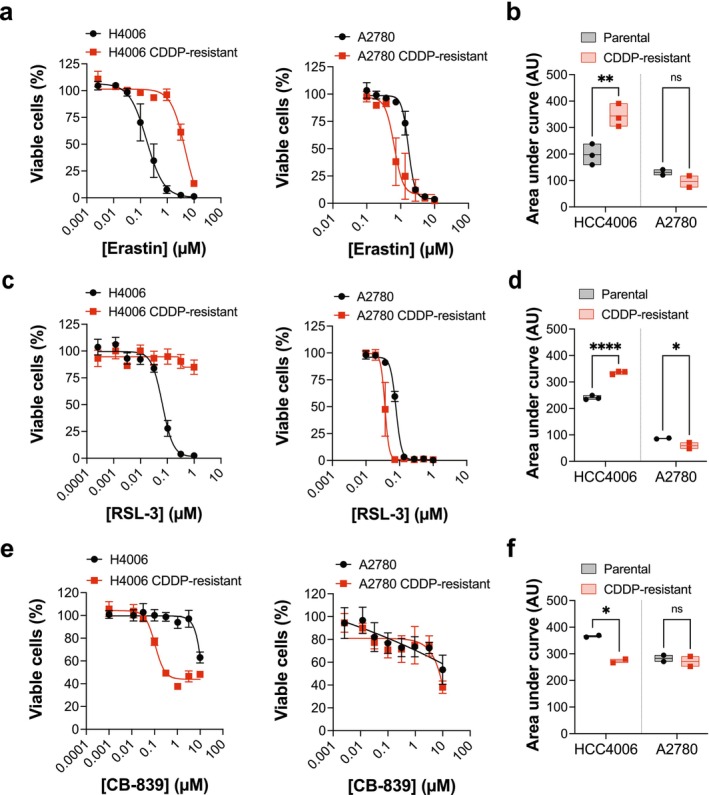
Cisplatin‐resistant cancer cells acquire differential sensitivity to ferroptosis inducers and to glutaminase inhibition. Parental and cisplatin (CDDP)‐resistant cell line pairs were exposed to a titration of the indicated compound (a,b, erastin; c,d, RSL‐3; e,f, CB‐839) and cell viability measured after 3 days via resazurin reduction, dose–response and area under the dose–response data shown. Mean values from 2 to 3 independent experiments (as indicated) plotted, with each experiment performed with technical duplicates. Error bars on dose–response data indicates SEM, box on area under dose–response data indicates mean and minimum/maximum. Ordinary one‐way ANOVA: *p < 0.05; **p < 0.01, ****p < 0.0001; ns, not significant. Drug titrations used—erastin, HCC4006 pair: 10, 3.2, 1, 0.32, 0.1, 0.03, 0.01, 0.003 μM; erastin A2780 pair: 10, 5.19, 2.72, 1.36, 0.72, 0.37, 0.19, 0.1 μM; RSL‐3, HCC4006 pair: 1, 0.32, 0.1, 0.03, 0.01, 0.003, 0.001, 0.0003 μM; RSL‐3 A2780 pair: 1, 0.52, 0.27, 0.14, 0.07, 0.04, 0.02, 0.01 μM; CB‐839, HCC4006 pair: 10, 3.2, 1, 0.32, 0.1, 0.03, 0.01, 0.001; CB‐839, A2780 pair: 10, 3.2, 1, 0.32, 0.1, 0.03, 0.01, 0.003.

We began by evaluating the cell line panel for sensitivity to erastin, targeting SLC7A11 [16], and RSL‐3, identified as targeting the lipid peroxidase GPX4 [17] that functions downstream of SLC7A11, although it should be noted that the selectivity of RSL‐3 was recently called into question [18]. Regardless, these targeted agents induce ferroptosis, an iron‐dependent cell death mechanism with broad implications for overcoming therapy resistance [19]. We observed that the HCC4006 cisplatin‐resistant cell line was cross‐resistant to erastin and RSL‐3 (Figure 2a–d), while a slight increased sensitivity was observed in the A2780‐cisplatin resistant cell model (Figure 2a–d).

CB‐839 (telaglenastat) is a potent and selective, orally bioavailable GLS1 inhibitor in advanced clinical testing [20, 21]. Treatment of the cell line panel with a titration of this compound revealed a striking sensitivity of the HCC4006 cisplatin‐resistant cancer cells, with the half maximal inhibitory concentration (IC_50_) value decreasing by several orders of magnitude compared to the parental counterpart (Figure 2e,f). In contrast, the A2780 cisplatin‐resistant cell model and parental counterpart showed no differential sensitivity (Figure 2e,f).

Taken together, these data demonstrate that acquired resistance to cisplatin in cancer cell models can result in differential sensitivity to targeted agents.

### 3.3 Acquired Resistance to Cisplatin Can Lead to Dependency on Extracellular Glutamine

**FIGURE 3:**
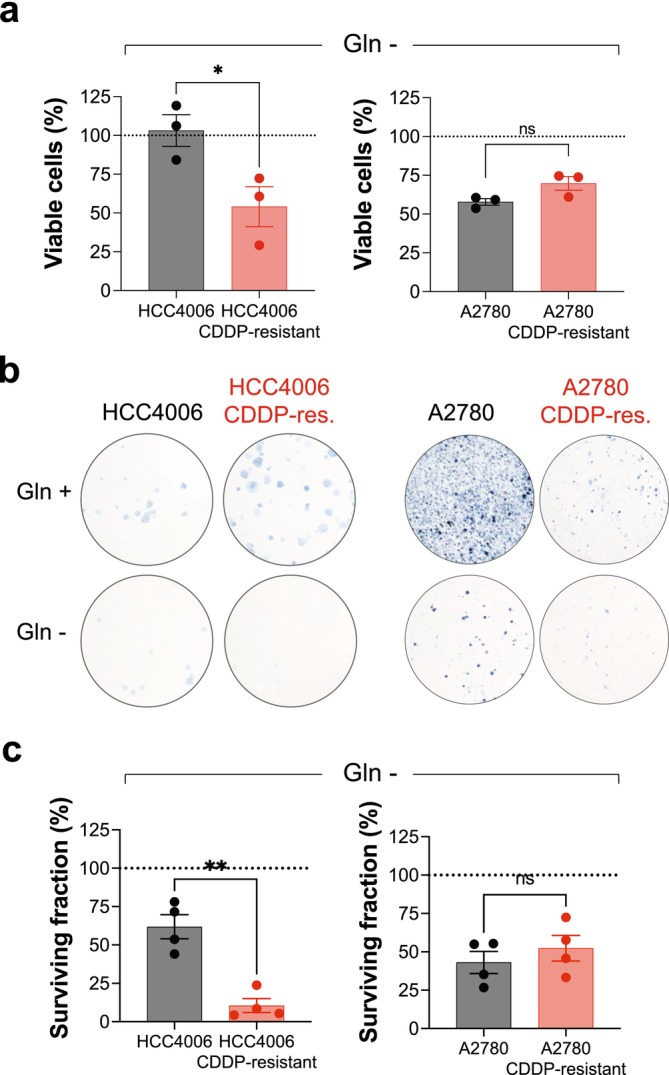
Acquired resistance to cisplatin can lead to a dependency upon extracellular glutamine in cell line models. (a) Parental and cisplatin (CDDP)‐resistant cell line pairs were cultured in complete media or media lacking glutamine (Gln –) for 2 days prior to resazurin reduction assay. Proportion of viable cells in the absence of glutamine relative to cells cultured in complete media (dashed line) plotted. Mean values from three independent experiments shown with each experiment performed with a minimum of six technical replicates. Bars and error bars indicate mean and SEM. Unpaired two‐tailed *t* test: *p < 0.05; ns, not significant. (b,c) Parental and CDDP‐resistant cell line pairs were cultured in complete media (Gln +) or media lacking glutamine (Gln –) in a colony outgrowth assay before fixation and staining. Representative images shown in (b) and surviving fraction relative to complete media control (dashed line) plotted in (c). Mean values from four independent experiments shown with each experiment performed with technical duplicates. Bars and error bars indicate mean and SEM. Unpaired two‐tailed *t* test: **p < 0.01; ns, not significant.

In search of a common vulnerability of cisplatin‐resistant cancer cells, we next interrogated whether these cells had increased dependence upon glutamine, given the altered expression of glutamine metabolic enzymes we had observed (Figure 1c,d). We plated cells in complete medium in the presence or absence of glutamine and, following 2 days, measured the remaining viable cells. While the parental HCC4006 cells showed no dependence upon extracellular glutamine, their cisplatin‐resistant derivative did show significantly more dependence upon this amino acid for proliferation (Figure 3a). In contrast, both the parental and cisplatin‐resistant A2780 cells were equally dependent upon extracellular glutamine for proliferation. We recapitulated both these results in colony outgrowth assays performed in the presence or absence of glutamine (Figure 3b,c).

**FIGURE 4:**
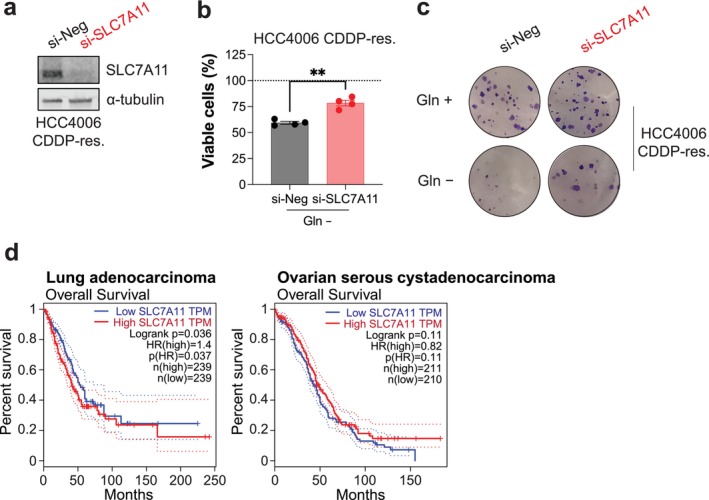
SLC7A11 expression contributes to glutamine dependence of cisplatin‐resistant lung adenocarcinoma cell model and correlates with overall survival in this malignancy. (a) HCC4006 cisplatin (CDDP)‐resistant cells were transfected with control (si‐Neg) or SLC7A11 targeting (si‐SLC7A11) siRNA and after 3 days, lysates prepared and analyzed by immunoblot with the indicated antibodies. A representative cropped blot from four independent experiments shown. (b) Cells as transfected in (a) were cultured in complete media or media lacking glutamine (Gln –) for 2 days before resazurin reduction assay. Proportion of viable cells in the absence of glutamine relative to cells cultured in complete media (dashed line) plotted. Mean values from four independent experiments shown with each experiment performed with a minimum of six technical replicates. Bars and error bars indicate mean and SEM. Unpaired two‐tailed *t* test: **p < 0.01. (c) Cells as transfected in (a) were cultured in complete media (Gln +) or media lacking glutamine (Gln –) in a colony outgrowth assay before fixation and staining. Representative images shown from two independent experiments shown. (d) Kaplan–Meier survival analysis was performed using the GEPIA platform for lung adenocarcinoma (LUAD) and ovarian serous cystadenocarcinoma (OV), stratifying patients by median *SLC7A11* expression (TPM, transcript per million). In LUAD (*n* = 478), high *SLC7A11* expression was associated with significantly worse overall survival (log‐rank *p* = 0.036; hazard ratio [HR] = 1.4; *p* (HR) = 0.037). In contrast, in OV (*n* = 421), *SLC7A11* expression was not significantly associated with survival (log‐rank *p* = 0.11; HR = 0.82; *p* (HR) = 0.11).

Seeking to understand the mechanistic basis of glutamine dependence, specifically in the HCC4006 cisplatin‐resistant model, we speculated it could be due to up‐regulation of the glutamate‐cystine antiporter SLC7A11 (Figure 1c,d). Thus, in the HCC4006 cisplatin‐resistant cell model, we depleted SLC7A11 protein using siRNA, confirmed by immunoblot analysis (Figure 4a), and subjected these cells to glutamine‐withdrawal before measuring remaining viable cells with orthogonal readouts. We observed that depletion of SLC7A11 partially reduced the glutamine‐dependence of the cisplatin‐resistant cancer model (Figure 4b,c), supporting that up‐regulation of SLC7A11 contributes to glutamine dependency. We next assessed the prognostic value of SLC7A11 expression in clinical lung and ovarian cancer cohorts, each comprising substantial numbers of cases with both low and high expression levels. SLC7A11 expression was significantly associated with poorer overall survival in lung cancer but showed no prognostic relevance in ovarian cancer (Figure 4d, Figure S1).

### 3.4 Acquired Resistance to Cisplatin Can Lead to Increased Sensitivity to ASNase

**FIGURE 5:**
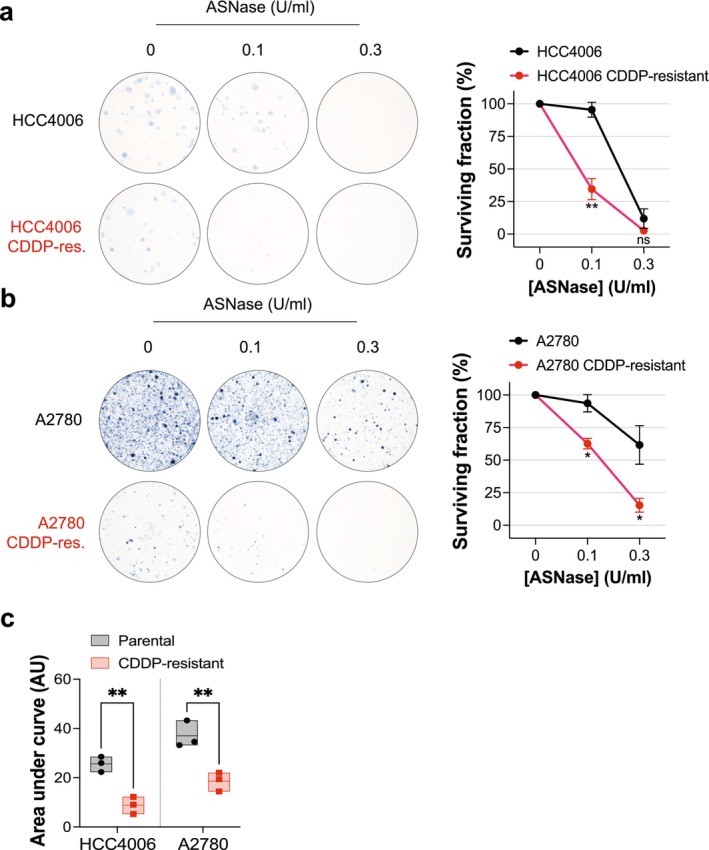
Cisplatin‐resistant cancer cells are sensitive to ASNase. (a,b) Parental and cisplatin (CDDP)‐resistant cell line pairs were cultured in complete media or media supplemented with asparaginase (ASNase; 0.3 or 0.1 U/mL) in a colony outgrowth assay, with drug supplementation replaced every 3–4 days, before fixation and staining. Representative images shown together with plot of surviving fraction relative to untreated control. Mean values from three independent experiments shown with each experiment performed with technical duplicates. Bars and error bars indicate mean and SEM. Unpaired two‐tailed *t* test comparing the parental and CDDP‐resistant cell line at each ASNase dose: *p < 0.05; **p < 0.01; ns, not significant. (c) Area under the dose–response data derived from (a,b). Mean values from three independent experiments, each performed with technical duplicates, shown; box indicates mean and minimum/maximum. Ordinary one‐way ANOVA: **p < 0.01.

Next, we evaluated the decades‐old anti‐leukemic therapy ASNase as a potential approach to target cisplatin‐resistant cancer cells. The clinical use of ASNase centers upon the enzymatic hydrolysis of extracellular asparagine, required for the proliferation of asparagine auxotroph leukemic cells, but ASNase also possesses glutaminase activity, and accordingly enzymatically depletes extracellular glutamine [22, 23]. Here, we interrogated the ability of ASNase to target cisplatin‐resistant cancer cells. Supplementation of cell medium with ASNase reduced the proportion of viable cells in a dose‐dependent manner in colony outgrowth assays (Figure 5a,b). Notably, both cisplatin‐resistant models showed elevated sensitivity compared to their parental counterparts, with the glutamine‐dependent HCC4006 cisplatin‐resistant model being the most sensitive (Figure 5a–c).

### 3.5 ASNase and Cisplatin Combine in an Additive Manner in Cisplatin‐Resistant Cancer Cells

**FIGURE 6:**
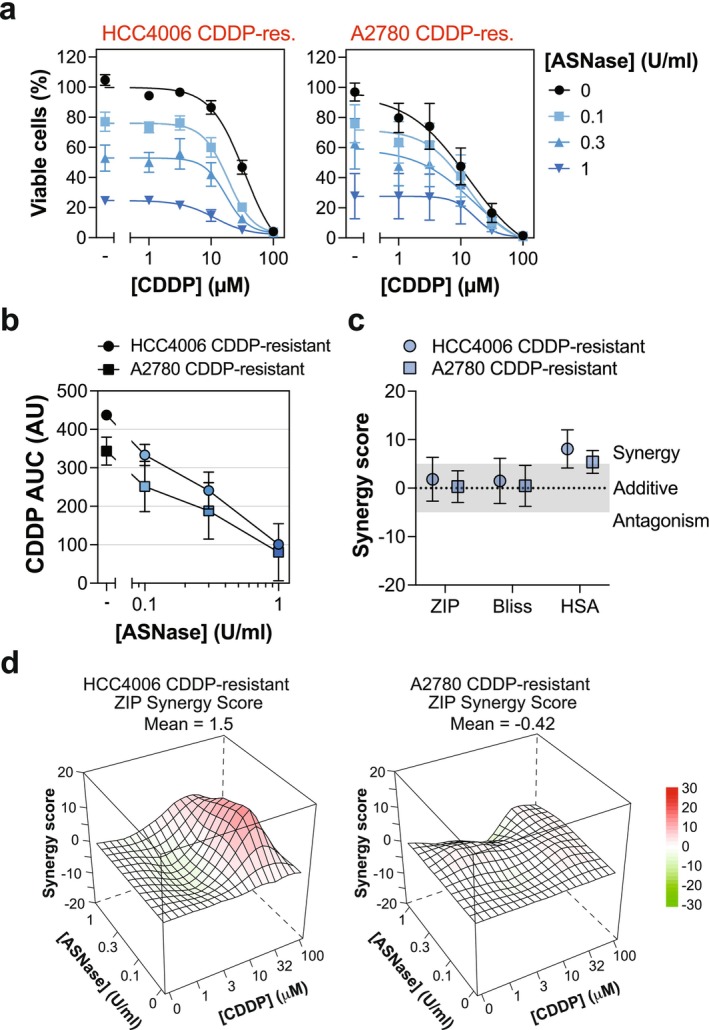
ASNase and cisplatin combination treatment in cisplatin‐resistant cancer cells results in additive inhibition of growth. (a) Cisplatin (CDDP)‐resistant cell lines were exposed to a CDDP (100, 32, 10, 3, 1 μM) vs. asparaginase (ASNase, 1, 0.3, 0.1 U/mL) dose matrix and cell viability measured after 3 days via resazurin reduction. Mean value from three independent experiments shown, each experiment performed with technical duplicates. Error bars indicate SEM. (b) CDDP area under the dose–response (AUC) as a function of ASNase concentration, derived from the data in (a). (c) Drug synergy plot for CDDP and ASNase combination in the indicated cell line with the indicated synergy model. Data derived from proliferation inhibition values in (a). Each dose–response matrix experiment was performed with technical duplicates and the mean values used to calculate the dose–response landscape from which the average synergy score derived. Data from three independent experiments shown, error bars indicate SEM. (d) Average synergy landscapes for the indicated cell lines shown using the ZIP synergy model, derived from data in (a).

Given ASNase is a cancer therapy in routine clinical practice and successfully targeted both cisplatin‐resistant cell models, we next sought to evaluate the applicability of this approach further and test whether it can be successfully combined with cisplatin therapy. We speculated that enzymatic depletion of extracellular glutamine by ASNase may re‐sensitize the cisplatin‐resistant cells to cisplatin. To assess this combination in depth, we seeded the cisplatin‐resistant HCC4006 and A2780 cancer cells upon dose matrices of cisplatin and ASNase and measured cell viability following a 3‐day treatment. ASNase dose‐dependently decreased the proportion of viable cells in combination with cisplatin (Figure 6a,b). The ability of ASNase to sensitize cells to cisplatin was noted in both A2780 and HCC4006 cisplatin‐resistant models, though more prominent in the glutamine‐dependent HCC4006 model (Figure 6a,b). We next used the data generated to assess the drug combination effect over the entire drug matrix by several synergy models, which overall showed the combination to be largely additive, with weak synergy observed with one synergy metric (Figure 6c). Inspection of the dose–response landscapes revealed a peak of synergy between ASNase and cisplatin in the HCC4006 cisplatin‐resistant model at select drug doses, which was absent in the A2780 cisplatin‐resistant model (Figure 6d). Overall, these data support that ASNase can be combined with cisplatin to target cisplatin‐resistant cancer cells.

## 4 Discussion

Despite many advances in treatment modalities, cisplatin‐containing chemotherapy regimens remain widely deployed in the treatment of solid malignancies. One of the main clinical limitations of this therapeutic agent is resistance. Thus, efforts are needed to identify treatment strategies to target cisplatin‐resistant cells. In the present study, we used cancer cell line pairs with acquired resistance to cisplatin, derived from a different solid malignancy in which cisplatin‐based therapy is used (lung and ovarian cancer). Using these models, we observed that acquired resistance to cisplatin can lead to altered expression of glutamine metabolic enzymes and differential sensitivity to targeted agents. We show that cisplatin‐resistant cancer cells can be dependent upon extracellular glutamine and subsequently identified ASNase therapy as a potential actionable strategy to target these cells.

Metabolic plasticity is a hallmark of cancer that is readily exploited in the acquirement of drug resistance, but this can also lead to collateral vulnerabilities of these drug‐resistant cells. In the case of acquired resistance to platinum therapy, rewiring of glutamine metabolism can lead to a dependence upon extracellular glutamine, which has been previously documented in cell models [24, 25, 26, 27, 28, 29, 30]. Furthermore, cisplatin‐resistant tumors grafted in mice have also been shown to be selectively sensitive to nutrient deprivation through repeated fasting cycles compared to cisplatin‐sensitive tumors [26], and high GLS1 expression has been correlated with reduced survival of ovarian cancer patients in several independent cohorts [24]. Here, we show that a cisplatin‐resistant experimental model, the lung HCC4006 cell line, also acquired dependence upon extracellular glutamine to sustain cell proliferation. This glutamine addiction could be mechanistically linked to up‐regulation of the glutamate‐cystine antiporter SLC7A11, as SLC7A11 protein level was higher in this model and depletion via siRNA could reduce the dependence upon extracellular glutamine. This mechanism is distinct from previous reports documenting glutamine addiction in cisplatin‐resistant cell models, which thus far have been attributed to elevated expression of ASCT2 and/or GLS1 [24, 28, 30], and promoter methylation‐mediated downregulation of GS [25].

Elevated SLC7A11 has been documented in cisplatin‐resistant cell lines [27, 31, 32, 33], patient‐derived material ex vivo [34], and tumors in vivo [32, 33], and in experiments in pre‐clinical models, has been demonstrated to contribute to cisplatin resistance via RNAi or targeting with small molecules [27, 31, 32, 33]. Furthermore, in the case of bladder cancer [32] and gastric cancer [33], high SLC7A11 expression correlates with poorer survival in cisplatin‐treated patients. In our study, we identify high SLC7A11 expression to significantly associate with poorer overall survival in lung but not ovarian cancer. We hypothesize that the elevated expression of SLC7A11 we observe in our model seeks to fuel GSH biosynthesis, to facilitate cisplatin resistance, which is consistent with earlier studies [27, 31, 32, 33]. But a consequence of increased cystine import to fuel GSH biosynthesis is increased glutamate export, which in turn renders this cell model dependent upon glutamine. This is also supported by literature outside the context of chemoresistance that has shown that SLC7A11 can dictate glutamine dependence in cell models [35, 36, 37, 38], and is supported in our study by the hypersensitivity of HCC4006 cisplatin‐resistant cells, with high SLC7A11 expression, to GLS1 inhibitor CB‐839, which indicates the elevated demand for glutamine is to generate glutamate. With regard to the A2780 cisplatin‐resistant model, although we observed reduced expression of GS, as previously reported [25], we did not see increased dependence of this model upon glutamine for cell proliferation. Instead, we observed that both the parental cell line and the cisplatin‐resistant derivative equally required glutamine for proliferation. The underlying mechanism in this case remains unclear.

In this study, we sought therapeutic approaches to target cisplatin‐resistant cells. Up‐regulation of a target can have differential effects upon cell killing through pharmacological inhibition of that same target. For instance, up‐regulation can indicate increased dependence of cells and thus enhanced sensitivity, or conversely, up‐regulation can also result in therapy resistance, given the higher levels of the target to be inhibited. We first evaluated experimental molecules known to induce ferroptosis that directly target SLC7A11 or downstream processes, as SLC7A11 was up‐regulated in the HCC4006 cisplatin‐resistant model. We observed that these cells became cross‐resistant to ferroptosis inducers erastin and RSL‐3, consistent with over‐expression studies outside the context of chemoresistance [39, 40], likely reflecting increased intracellular GSH levels, mediated by elevated SLC7A11, that would buffer against ferroptotic cell death. Altogether, these data indicate exploiting ferroptosis, and specifically SLC7A11 in cells with high SLC7A11 expression, as a means to kill cisplatin‐resistant cells may not be a viable strategy. However, in the A2780 cisplatin‐resistant model that did not up‐regulate SLC7A11, these agents were slightly more effective. Next, we evaluated the GLS1 allosteric inhibitor CB‐839 (telaglenastat) and observed striking sensitivity in specifically the HCC4006 cisplatin‐resistant model with high SLC7A11 expression, consistent with an earlier report demonstrating SLC7A11 activity can drive CB‐839 sensitivity [36]. This highlights a promising context to further investigate the use of this molecule, particularly in lung cancer in which we observed high SLC7A11 expression significantly associates with worse overall survival. In contrast, the A2780 cisplatin‐resistant line showed no significant difference in CB‐839 sensitivity compared to its parental counterpart. Altogether, these data highlight the difficulty in finding agents to robustly target cancer cells with acquired resistance to cisplatin.

ASNase is a clinically approved therapy which was the first to exploit a metabolic dependency in cancer cells, specifically the dependence of acute lymphoblastic leukemia (ALL) cells upon circulating asparagine. Here, we observed that cisplatin‐resistant cancer cells are more sensitive to ASNase, and unlike the targeted ferroptosis inducers and glutaminase inhibitor, ASNase sensitivity was consistent between the cisplatin‐resistant models, although elevated in the glutamine‐dependent HCC4006 model. Furthermore, ASNase appeared to re‐sensitize these models to cisplatin. We speculate the reason for this is that ASNase also possesses glutaminase activity and can thus deplete both asparagine and glutamine, which has been documented in plasma [22, 23]. We hypothesize the enzymatic depletion of glutamine would stifle GSH production, required to detoxify aquated cisplatin. The glutaminase activity may also be the reason for enhanced monotherapy sensitivity in the glutamine‐addicted HCC4006 model and potentially highlights the utility of elevated SLC7A11 expression in predicting response. However, given ASNase monotherapy was more effective in both cisplatin‐resistant models compared to parental counterparts—including the A2780 model which was not more dependent upon glutamine—this implies cisplatin‐resistant cells may have increased reliance upon extracellular asparagine also. There is precedent for cancer cells outside of ALL to have dependency upon asparagine, which can arise by multiple mechanisms [41]. The precise mechanism in this case remains to be investigated.

Outside the context of chemoresistance, there are several examples of exploiting the glutaminase activity of ASNase for targeting glutamine‐dependent cancer cells, including both solid [35, 42] and hematological cancer‐derived models [43, 44, 45]. Although glutamine is highly abundant in plasma [9], ASNase therapy can substantially deplete the level of this amino acid [23], and the glutaminase activity of ASNase is understood to contribute to the anti‐leukemic activity but is also considered to be a source of toxicity [23], and deciphering the relative contributions is an active area of research [46]. This could be one note of caution with regards to the repurposing of ASNase therapy against solid malignancies, particularly in adults. While ASNase therapy is routinely deployed successfully in the treatment of children with ALL, toxicities in adults can be limiting, and this has been observed in several trials against solid malignancies [47, 48]. Thus, studies to understand and overcome these limitations of ASNase therapy in solid malignancies are an ongoing research area [49, 50]. Nevertheless, repurposing ASNase therapy or the development of a specific glutaminase therapy [51, 52] to exploit glutamine addiction and/or re‐sensitize to cisplatin could be a promising approach. It should be noted that, relevant to the combination of ASNase with cisplatin resulting in an additive inhibition of proliferation in the cisplatin‐resistant models in our study, additive drug combinations can be curative [53].

## 5 Conclusion

In conclusion, in the present study, we provide evidence that repurposing of the decades‐old anti‐leukemic therapy ASNase could be a promising approach to target cisplatin‐resistant cells and possibly re‐sensitize to cisplatin. An important limitation of our study is the small number of cell models characterized, and future studies should expand upon this together with interrogating more complex pre‐clinical models of chemoresistance.

## Author Contributions

Conceptualization: Jiantao Wang and Sean G Rudd. Methodology: Jiantao Wang, Nasim Pouryaghoub, Robert Strauss, Jiri Bartek, Si Min Zhang and Sean G Rudd. Investigation: Jiantao Wang, Nasim Pouryaghoub, Robert Strauss, Jiri Bartek, Si Min Zhang and Sean G Rudd. Writing – original draft: Jiantao Wang and Sean G Rudd. Writing – reviewing and editing: Jiantao Wang, Jiri Bartek, Si Min Zhang, and Sean G Rudd. Visualization: Jiantao Wang, Robert Strauss, Si Min Zhang, and Sean G Rudd. Resources: Robert Strauss and Jiri Bartek. Funding acquisition: Jiri Bartek and Sean G Rudd. Supervision: Jiri Bartek, Si Min Zhang, and Sean G Rudd.

## Conflicts of Interest

The authors declare no conflicts of interest.

## Supporting information


**Figure S1:** Kaplan–Meier survival analyses were performed using the KM‐plotter platform for lung and ovarian cancer—as detailed in Materials and Methods—stratifying patients by median *SLC7A11* expression. In lung cancer (*n* = 2166), high *SLC7A11* expression was associated with significantly worse overall survival (log‐rank *p* = 0.019; hazard ratio [HR] = 1.15). In contrast, in ovarian cancer (*n* = 1435), SLC7A11 expression was not significantly associated with survival (log‐rank *p* = 0.27; HR = 0.93).

## Data Availability

Data can be made available by the authors upon request.
