# Supplementary material for: Repurposing Asparaginase Therapy to Target Cisplatin‐Resistant Cancer Cells
Source: Fundam Clin Pharmacol. 2025 Aug 10;39(5):e70044. doi: 10.1111/fcp.70044 (PMC12335911; doi:10.1111/fcp.70044)
Supplement: Supplementary file 1 — Figure S1: Kaplan–Meier survival analyses were performed using the KM‐plotter platform for lung and ovarian cancer—as detailed in Materials and Methods—stratifying patients by median SLC7A11 expression. In lung cancer (n = 2166), high SLC7A11 expression was associated with significantly worse overall survival (log‐rank p = 0.019; hazard ratio [HR] = 1.15). In contrast, in ovarian cancer (n = 1435), SLC7A11 expression was not significantly associated with survival (log‐rank p = 0.27; HR = 0.93). [file FCP-39-0-s001.pdf]

Lung cancer **Ovarian cancer**

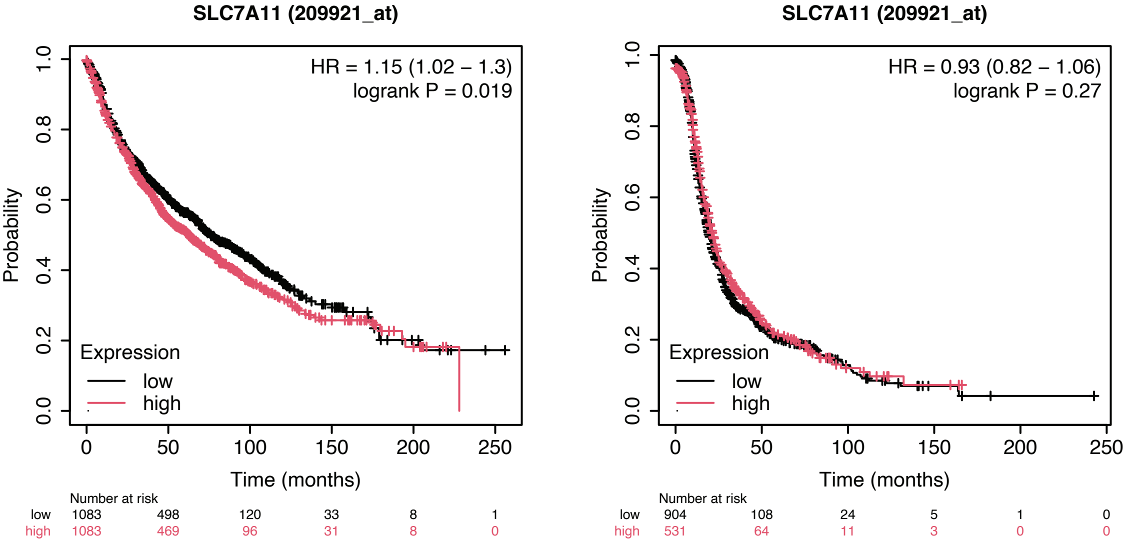

Figure S1. Kaplan–Meier survival analyses were performed using the KM-plotter platform for lung and ovarian cancer - as detailed in Materials and Methods - stratifying patients by median *SLC7A11* expression. In lung cancer (n = 2166), high *SLC7A11* expression was associated with significantly worse overall survival (log-rank p = 0.019; hazard ratio [HR] = 1.15). In contrast, in ovarian cancer (n = 1435), SLC7A11 expression was not significantly associated with survival (log-rank p = 0.27; HR = 0.93).
